# Detection of cow hind-leg activity during milking by using a 3-dimensional accelerometer attached to the milking cluster

**DOI:** 10.3168/jdsc.2020-0020

**Published:** 2021-01-22

**Authors:** C.M.C. Raoult, A.A. Margerit, S. Fricker, F.E. Blümel, P.E. Savary

**Affiliations:** 1 Centre for Proper Housing of Ruminants and Pigs, Federal Food Safety and Veterinary Office, Agroscope Tänikon, 8356 Ettenhausen, Switzerland; 2 Independent researcher, 77750 Saint-Cyr-sur-Morin, France; 3 Department of Livestock Systems Engineering, Institute of Agricultural Engineering, University of Hohenheim, 70593 Stuttgart, Germany; 4 Competitiveness and System Evaluation, Agroscope Tänikon, 8356 Ettenhausen, Switzerland

## Abstract

Hind-leg activity in dairy cows during milking is considered an indicator of a stressful situation or discomfort. Automatic detection of cow hind-leg activity during milking could be used to monitor deficiencies at the cow, milking machine, or human level. We assumed that the milking cluster follows cow hind-leg movements during milking. Therefore, the aim of our study was to estimate the ability of a 3-dimensional (3D) accelerometer attached to the claw's hook of the milking cluster to indirectly measure cow hind-leg activity during milking. The behavior of 45 dairy cows during 1 morning and 1 evening milking in a 2 × 3 auto tandem milking parlor was monitored by using direct visual observations and a 3D accelerometer attached directly to the hook of each milking claw. We recorded when hind-leg foot-lifting or stepping (undifferentiated) and kicking behaviors occurred. Detected activities were obtained after applying a standard deviation filter (over a sliding window of 1 s) on the x (left/right lateral) acceleration axis only. We set the detection threshold at 0.13 *g* (i.e., 1.27 m·s^−2^), based on the visual evaluation of 10 “reference” graphs (from 5 randomly chosen cows). Periods of activity were created for each observed or detected activity (and for consecutive activities that were less than 3 s apart) to take into account the milking cluster's rocking motion resulting from a single movement. Observed and detected periods of activity were considered matching when they occurred simultaneously (with a 2-s delay acceptance in the visual observations). In total, 472 periods of hind-leg activity were observed. The detection of hind-leg activity by using a 3D accelerometer attached to the milking cluster was found to have 69% sensitivity, 99% specificity, 75% positive predictive value, 98% negative predictive value, and an overall accuracy of 97%. On average, cows were observed being active 0.94 times per minute and being detected active 0.86 times per minute. The observed and detected periods of activity were found to be correlated (r = 0.657). Therefore, the use of a 3D accelerometer attached to the milking cluster seems promising for reliably estimating cow hind-leg activity during milking. Nevertheless, further investigations are necessary to clarify whether other factors can cause variations in milking cluster movements, regardless of cow hind-leg activity, and to generalize our method to better identify deficiencies in cow–machine–human interactions.

## Highlights

• Hind-leg activity during milking in cows is a good welfare indicator
• Cow hind-leg movements are transmitted to the milking claw
• 3D accelerometers attached to the milking claws accurately record cow hind-leg movements

## Graphical Abstract

**Figure:**
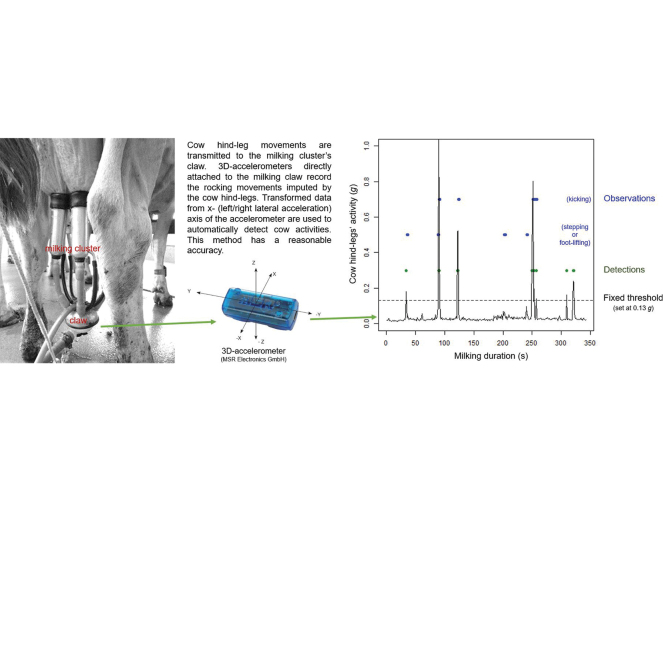
**Summary:** Hind-leg activity during milking, as an indicator of welfare impairment, was recorded on 45 dairy cows during 2 milkings by using direct visual observations and a 3-dimensional (3D) accelerometer attached directly to the hook of each milking claw. Our study shows that the use of a 3D accelerometer attached to the milking cluster is a promising method to reliably estimate cow hind-leg movements. An automatic and objective detection of foot-lifting, stepping, and kicking behaviors allows analysis of the causes and optimization of the milking process to improve animal welfare, cow (udder) health, and milking performance, as well as reduction of such potentially dangerous behaviors.

Daily milking is a common routine for dairy cows that involves a strong interaction between animal, human, and milking equipment. Cow behavioral changes during milking can serve to highlight “welfare problems relating to udder health [including teat condition], milking techniques [including milking machine configurations], skin lesions caused by ticks and quality of handling routines in the individual herd” (Rousing et al., 2004). Restlessness behavior during milking is considered an indicator of a stressful situation (Willis, 1983; Gygax et al., 2008), and an increase in kicks and steps might highlight cow discomfort (Penry, 2018). In particular, Penry (2018) proposed to integrate these behavioral changes into an investigation protocol, including milking time observations such as in the Lactation Technote 13 of Dairy Australia (2003), so that when kicks and steps reached a certain threshold, it would trigger investigations on causes. Stepping, flinching, foot-lifting, and kicking behaviors during milking occur for multiple reasons, such as deficiencies in the milking machine or husbandry conditions in the milking parlor (e.g., aversion to the human handler; Rushen et al., 1999; Rousing et al., 2004). In general, technical deficiencies of the milking machine, such as over-milking (Cerqueira et al., 2017), high system vacuum levels, vacuum drops at the teat end (Besier et al., 2016), or high vacuum level in the mouthpiece chamber of the liner (Newman et al., 1991; Penry et al., 2017), have a detrimental effect on the teat (congestion, ringing at the teat base, lesions) and udder tissue conditions, which can lead to pain as well as defensive reactions and restlessness in cows. Intrinsic cow factors, such as parity or udder health, can also have an influence. Multiparous cows seem to kick less (Cerqueira et al., 2017) and step more (Gygax et al., 2008; Cerqueira et al., 2017) than primiparous cows; however, Rousing et al. (2004) observed that high-yielding cows older than parity 4 stepped less than did younger cows. Cows with an SCC >200,000 cells/mL (Gygax et al., 2008; Cerqueira et al., 2017) or with teat lesions (Rousing et al., 2004) were also found to kick more during milking. To limit the occurrence of these behaviors, they should first be detected objectively and easily. Behavioral parameters are often monitored by means of video recordings or direct visual observations. However, these methods are time consuming and labor intensive, require trained observers (Winckler and Willen, 2001; Rushen et al., 2012), and cover only a short period of time. Automated data recording using, for example, a wireless system can thus be seen as a solution (Müller and Schrader, 2003). In particular, accelerometers have been found to record animal (locomotor) activities and postures (Moreau et al., 2009; de Passillé et al., 2010; Rushen and de Passillé, 2012) with reasonable accuracy compared with direct observations or videos (Martiskainen et al., 2009). Usually, wireless sensors are attached directly to the animals; for instance, to one hind-leg (Rushen and de Passillé, 2012), or mounted on a harness or collar (Moreau et al., 2009). However, to avoid attaching measurement technology directly to the animals and to reduce the number of sensors needed to measure each cow's hind-leg movements during milking independently of the herd size, in this study we used 3-dimensional (**3D**) accelerometers freely suspended on the milking cluster claw hooks. We assumed that activity of the hind-legs would be transmitted with sufficient accuracy to the milking cluster (i.e., the milking unit swaying with the hind-leg movements).

To test this hypothesis, we monitored 45 dairy cows (28 Brown Swiss and 17 Red Holstein × Swiss Fleckvieh; 8 cows <100 DIM, 19 cows 100–200 DIM, and 18 cows >200 DIM; 18 primiparous and 27 multiparous) during 1 morning (0430 h) and 1 evening (1630 h) milking in a 2 × 3 auto tandem milking parlor (GEA Farm Technologies GmbH, Bönen, Germany) in March 2014 at the Agroscope Research Station in Tänikon, Switzerland. The milking machine system vacuum was set at 38 kPa, the 2 × 2 (alternating) pulsation rate at 60 cycles/min, and the pulsator ratio at 60:40 (with the a-, b-, c-, and d-phases set to 140, 460, 70, and 330 ms, respectively). To avoid incomplete milkings, the automatic stripping system Finilactor (GEA Farm Technologies GmbH, Bönen, Germany) was used. This system, integrated in the milking arm, applies a slight pressure on the claw when the milk flow drops below the threshold value of 0.8 kg/min and until the cluster is automatically removed at a flow threshold of 0.3 kg/min. Milking was performed by the same person using consistent preparation procedures (fore-stripping, pre-dipping, and drying). The udder preparation time before attachment of the milking unit took approximately 1 min. After milking unit attachment, the cows were automatically stimulated with a pulsation of 200 pulses per second for 30 to 50 s, depending on the lactation stage (as standard procedure at the Research Station). During milking, we recorded the cows' foot-lifting or stepping (undifferentiated) and kicking behaviors (based on Kutzer et al., 2015) in both hind-legs by direct visual observations [sampling rate at 1 Hz; using an appropriate spreadsheet (Excel 2010, Microsoft Corp., Redmond, WA) on a touch pad time-synchronized with the accelerometers]. Visually observed foot-lifting or stepping were indifferently defined as a foot elevated less than 15 cm off the ground (i.e., with or without weight displacement; contrary to the definitions of Kutzer et al., 2015), whereas kicking in any direction corresponded to a hoof lifted at least 15 cm. The logic behind this simplified categorization was to allow comparison with accelerometer measurements, for which a weight displacement could not be assessed. These same behaviors were also recorded by using a 3D accelerometer (MSR145 data logger, 20 × 15 × 52 mm, ~0.016 kg; MSR Electronics GmbH, Seuzach, Switzerland) attached to the claw's hook of each of the 6 milking clusters (GEA Classic 300 milking cluster, GEA Farm Technologies GmbH). We programmed and synchronized all the loggers identically and transferred the MSR data using the software MSR 5.24.02 (MSR Electronics GmbH). We recorded the acceleration on the x- (left/right lateral acceleration, perpendicular to the cow body axis), y- (forward/backward acceleration, parallel to the cow body axis), and z- (vertical acceleration) axes with an accuracy of ± 0.15 *g*, a resolution of ± 0.03 *g*, and a range of ±15 *g* (where 1 *g* = 9.81 m·s^−2^) and set the sampling rate at 50 Hz. The aim of this work was to test whether hind-leg activity during milking could be detected indirectly by using a 3D accelerometer attached to the claw's hook of the milking cluster.

Data processing and statistical analyses were performed in R version 3.6.1. (R Core Team, 2019). We imported the MSR data and direct visual observations into R, so that the milking place, cow number, time of day (morning or evening milking), start and stop of the milking, and observed activities coincided. We observed from 10 reference graphs (described later) that the activity of the hind-legs creates a left–right rocking motion of the milking cluster (i.e., on the x acceleration axis) with enough intensity to be detected by the accelerometer (see Figure 1). Data from the y acceleration axis provided a similar response curve to that of the x acceleration axis and did not improve the detection accuracy, whereas data from the z- acceleration axis led to detection of more activities than were visually observed. Therefore, to detect hind-leg activities, we used only the x acceleration axis values. Additionally, to remove small variations but preserve large acceleration variations, we applied a standard deviation filter over a sliding window of 1 s, meaning that we calculated the absolute value of the difference between the value measured and the rolling mean of the values measured during the previous second (at a sampling rate of 50 Hz, 1 s corresponds to 50 values). Furthermore, we wanted to avoid detection of cluster movements unrelated to hind-leg activities as measured with the y- and z-axes. The activity detection threshold was defined at 0.13 *g* (i.e., 1.27 m·s^−2^), based on the visual evaluations of 10 graphs from 1 morning and 1 evening milking of 5 randomly selected cows, for which we also had observed activities (referred to as “reference” graphs; for an example, see Figure 1). These cows were excluded from the remaining analyses, as were data from the morning milking of 1 additional cow because its direct visual observations were incomplete. Therefore, statistical analyses were performed on 79 milkings from 40 cows. The aim of this study was to determine whether observed hind-leg activities could be estimated by using a 3D accelerometer directly attached to the milking cluster rather than to differentiate kicks from steps. Thus, we considered kicks and steps undifferentiated. A period of activity was created for each activity visually observed, starting at the beginning of the observation and ending at the end of the observation and therefore varying in length (e.g., 1 s, 2 s, 3 s, 4 s or more). Consecutive activities that were less than 3 s apart were merged as a single period of activity (starting at the beginning of the first observation and ending at the end of the second or last observation). Periods of activity were created in the same way for the detected activities (acceleration peaks >0.13 *g*). We did so to account for the rocking motion of the milking cluster (the 3-s period being chosen based on visual observations), which will produce several acceleration peaks for a single foot movement, but also because cows often step repeatedly in short time intervals. Matching observed and detected periods of activity were considered when they occurred simultaneously, with an acceptance of up to 2-s delay in the visual observations. As done by Röttgen et al. (2020), we calculated for each cow and milking the number of true positive (**TP**; when a period of activity was observed and detected), false positive (**FP**; when a period of activity was not observed but detected), false negative (**FN**; when a period of activity was observed but not detected), and true negative (**TN**; when a period of activity was neither observed nor detected) periods of activity to determine the following indices:

$$\text{Sensitivity}=\frac{\text{TP}}{\text{TP}+\text{FN}}=\frac{\text{true test positive}}{\text{total observations of period of activity}};$$

$$\text{Specificity}=\frac{\text{TN}}{\text{TN}+\text{FP}}=\frac{\text{true test negative}}{\text{total observations of no period of activity}};$$

$$\text{Positive predictive value}=\frac{\text{TP}}{\text{TP}+\text{FP}}=\frac{\text{true test positive}}{\text{total detections of period of activity}};$$

$$\text{Negative predictive value}=\frac{\text{TN}}{\text{TN}+\text{FN}}=\frac{\text{true test negative}}{\text{total detections of no period of activity}};$$

$$\text{Prevalence}=\frac{\text{TP}+\text{FN}}{\text{TP}+\text{TN}+\text{FP}+\text{FN}}=\frac{\text{total observations of period of activity}}{\text{total observations and detections}};$$

$$\text{Accuracy}=\frac{\text{TP}+\text{TN}}{\text{TP}+\text{TN}+\text{FP}+\text{FN}}=\frac{\text{true test positive and negative}}{\text{total observations and detections}}.$$

**Figure 1:**
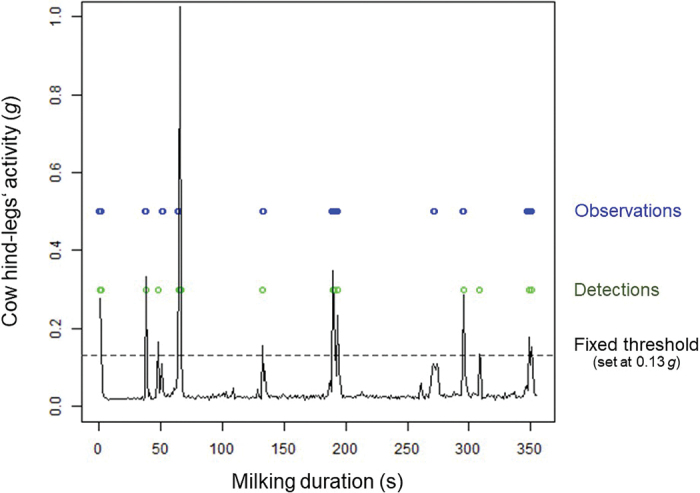
Example of cow hind-legs activity during one milking reported based on the x-axis (left/right lateral acceleration) values of the 3-dimensional accelerometer after a standard deviation filter over a sliding window of 1 s was applied. Visual observations of the foot-lifting (or stepping) and kicking behaviors are in blue and the detections (when higher than the threshold) in green. The dotted black line symbolizes the threshold set at 0.13 *g*.

To calculate the TN (i.e., periods of inactivity), we had to harmonize the duration of the different periods. As such, to avoid comparing short periods of activity (e.g., of 3 s) with longer periods of inactivity (e.g., of 30 s or more), we divided the inactivity period into smaller periods of 3 s, so that the longer the period of inactivity detected, the higher the number of TN. Sections of 3 s were chosen to coincide with the approximate duration of an activity, thus best reflecting the specificity of our detection method. We also defined the average number of periods of activity per cow per milking per minute and performed a Pearson correlation analysis between the observed and detected periods of activity per cow per milking per minute.

In total, 472 periods of hind-leg activity were observed (Table 1) with a prevalence of 4.82% for having a period of activity. The automated detection of the cows' hind-leg activity, by using a 3D accelerometer attached to the milking cluster, was found to have a sensitivity of 68.86% (i.e., the percentage of observed activities that were detected), a specificity of 98.85%, including 107 activities that were detected but not observed, a positive predictive value (or precision) of 75.23%, a negative predictive value of 98.43%, and an accuracy of 97.41% (Table 1).

**Table 1:** Overview of the number of observed and detected periods of cow hind-leg activity and the derived indices

	Observed activity	No observed activity	
Detected activity	True positive	False positive	Positive predictive value
	325	107	75.23%
No detected activity	False negative	True negative	Negative predictive value
	147	9,221	98.43%
	Sensitivity	Specificity	Accuracy
	68.86%	98.85%	97.41%

The milking lasted on average 6.14 min, during which the cows were observed being active 0.94 times per minute (including 0.23 kicks per minute) and being detected active 0.86 times per minute. The observed and automatically detected periods of activity were found to be correlated (r = 0.657; *P* < 0.001; Figure 2).

**Figure 2:**
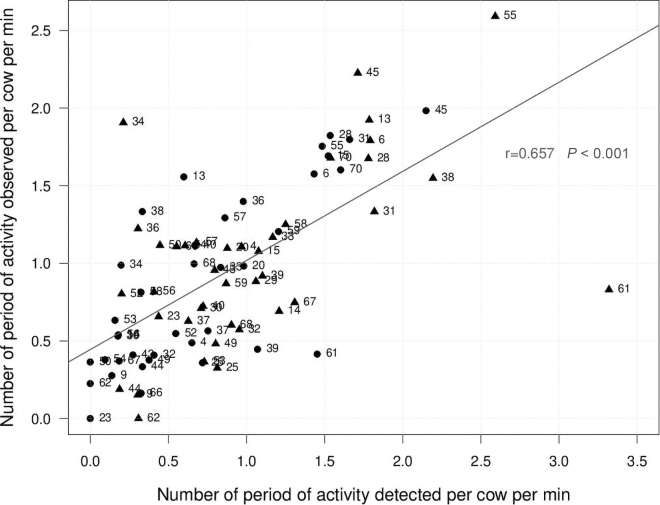
Correlation between the number of observed and detected periods of activity per cow per minute. Black points and triangles represent the cows (each cow having a different number) during their morning and evening milking, respectively. The thin dark gray line symbolizes the Pearson correlation.

In our study, we found that hind-leg movements of cows during milking are transferred to the milking cluster, creating an acceleration measurable by the sensor (as it is graphically visible once the standard deviation filter was applied on the x acceleration axis; for an example, see the graphical abstract). Here, these movements could be detected with reasonable accuracy by using a 3D accelerometer attached to the claw's hook of the milking cluster (and a threshold set at 0.13 *g*; i.e., 1.27 m·s^−2^). The results show that our automated detection method was promising in regards to its sensitivity, high specificity, positive predictive value (three-fourths of the periods of activity detected were true periods of activity), and high negative predictive value. The predictive values obtained here are particularly meaningful because of the low prevalence rate in the population tested. In fact, predictive values are influenced by prevalence as follows: as prevalence increases, the positive predictive value increases, and negative predictive value decreases (Parikh et al., 2008).

Different factors could explain the over- and under-detections of the hind-leg activity of cows during milking. Dynamic conditions occur in the milking machine (Besier et al., 2016), such as mechanical liner movements (i.e., pulsation) and pressure differences (i.e., vacuum fluctuations), which could set the cluster in motion and lead to over-detection. The effect of different pulsation settings and milk flow levels on cluster movements could be the focus of further investigations. However, the accelerometer was positioned on the claw's hook of the milking cluster, which (in an unpublished pilot study) was found to be the location with the lowest disturbing fluctuations produced by the milking tube. Other aspects that could affect the transmission of hind-leg movements to the cluster are the cow teat and udder conformations, which can be very different within a herd (Zwertvaegher et al., 2012).

In addition, we defined a fixed detection threshold for all cows tested, based on the visual observation of the data of 5 randomly selected cows. The difficulty we faced was in defining a threshold that would detect all movements (regardless of their type—foot-lifting, stepping, or kicking) but would not be too low to avoid noise detection. However, the cows might have had different baseline noise activities (e.g., breath, heart rate, rumen activity) and temperament (e.g., more or less active, strength variation). This threshold was therefore not optimized for each individual cow.

Because we used a high sampling rate (i.e., 50 Hz) which is known to increase accuracy of detection, we looked at the acceleration (passed through a standard deviation filter) on the x- (left–right lateral) axis only and not, for example, the summed accelerations of the 3 axes (Rushen and de Passillé, 2012). In a previous study, Luu et al. (2013) found that measuring acceleration in the vertical axis only did not seem to markedly reduce the accuracy of the gaits estimates. Looking at the accelerations on the y- and z-axes (or summed acceleration) here would have added noise unrelated to the hind-leg movements.

In the current study, one observer made all the direct visual observations due to space restrictions in the milking parlor. Although we could argue that using video recordings would have been more reliable to measure hind-leg activities during milking, we believe that the observer was less likely to miss hind-leg activities because of having greater adaptability to the situation (e.g., the observer could move away from the milker).

We studied only 1 herd of 40 cows during 2 milkings. To further test our method (and threshold), we should thus prove its accuracy with data from other herds, milking system types, and claw types. Because of the lack of comparable data, we could not verify whether cow characteristics (e.g., the cow's milk flow, lactation stage, parity), the time point of the milking, or the milking machine (e.g., claws, sleeves) and its calibration (e.g., vacuum level, pulsation types) had an effect on the sensitivity and specificity of our automatic detection method. For example, Bayer (1969) reported that cows have a lower heart rate in the morning, which could suggest behavioral differences (Wenzel et al., 2003) in the evening milking when cows are less relaxed.

Finally, comparing the number of cow hind-leg activities occurring during milking between our study and previous works was difficult because different activities were recorded using different definitions and were performed in different milking systems (e.g., automatic milking system, auto tandem milking parlor, or herringbone milking parlor). However, the milking parlor type does not seem to play a major role in the occurrence of stepping and kicking during milking (Gómez et al., 2017), although milking duration in an automatic milking system is, on average, shorter than in other systems. In our study, we counted the number of periods of activity observed per minute (i.e., all foot-lifting, stepping, and kicking behaviors combined), whereas previous studies recorded either the frequency or number of occurrences per milking of single steps, kicks, foot-liftings, or steps and kicks (see the summary table; Table 2). We might therefore have found fewer periods of activity than previous studies found steps and kicks. The 0.94 periods of activity per minute, including few kicks (i.e., 0.23 kicks per minute), that we observed in an auto tandem milking parlor seem consistent with the available literature. The defined periods of activity (merging single consecutive activities that are less than 3 s apart) appear to be an accurate estimate.

**Table 2:** Frequency of cows' hind-leg activity during milking by study and type of milking system

Study	Milking system type^1^	Foot-lifting and stepping rate (min^−1^)	Kicking rate (min^−1^)	Activity rate (min^−1^)
Hopster et al., 2002^2^	AMS	1.13^3^	No data	1.13
	ATM	1.11^3^	No data	1.11
Wenzel et al., 2003	AMS	0.61^3^	0.14	0.75
	ATM	0.08^3^	0.07	0.15
Hagen et al., 2004	AMS	1.01^3^	0.07	1.08
	HM	2.22^3^	0.29	2.50
Pastell et al., 2006^4^, ^5^	AMS	1.84		1.84
Gygax et al., 2008^6^	AMS 1	0.54	No data	0.54
	AMS 2	0.81	No data	0.81
	ATM	0.75	No data	0.75
Kutzer et al., 2015^7^	HM/ATM	0.20	0.30	0.50
Present study (direct visual observations)	ATM	0.71^3^	0.23	0.94

1 AMS, automatic milking system; ATM, auto tandem milking parlor; HM, herringbone milking parlor.

2 Kicking was not recorded.

3 Foot-lifting and stepping were not distinguished.

4 Results of healthy cows only.

5 Frequency of the foot-lifting, stepping, and kicking combined and calculated with an assumed milking duration of 7.16 min (based on milking duration in Wenzel et al., 2003).

6 Although the occurrence of kicking was recorded, it was not available in the article.

7 Behavior of heifers only.

Here, we highlight the potential of our method to estimate the number of periods of activity regardless of the cow activity (Figure 2). The use of a 3D accelerometer attached to the claw's hook of the milking cluster seems promising for reliably, objectively, automatically, and indirectly estimating cow hind-leg activities during milking. However, the results of this study show the limitation of using a fixed threshold, which can lead to both over- and under-estimation of movements, as shown in Figure 2. A cow- (or even milking-) individualized threshold would thus probably optimize general detection accuracy. Accurate detection of foot-lifting (or stepping) and kicking behaviors during milking is a prerequisite if using this method in an automated monitoring tool of the milking process, milking machine configuration, and cow (udder) health. Further investigations are needed to clarify whether other factors can cause milking cluster movement variations regardless of the cows' hind-leg activity, as well as to generalize and validate the method, in other herds, milking systems, and claw types.
